# Primary sclerosing cholangitis and pancreatic cancer: A retrospective cohort study of United States veterans

**DOI:** 10.3389/fgstr.2022.1076788

**Published:** 2023-01-24

**Authors:** Anita Nguyen, Babak Torabi Sagvand, Madeline Alizadeh, Cydney Nguyen, William Scott, Erik C. von Rosenvinge

**Affiliations:** ^1^ Department of Veterans Affairs, Veterans Affairs (VA) Maryland Health Care System, Baltimore, MD, United States; ^2^ Department of Medicine, University of Maryland Medical Center, Baltimore, MD, United States; ^3^ Department of Medicine, Division of Gastroenterology and Hepatology, University of Maryland Medical Center, Baltimore, MD, United States; ^4^ Institute for Genome Sciences, University of Maryland School of Medicine, Baltimore, MD, United States

**Keywords:** veteran, primary sclerosing cholangitis, PSC, pancreatic cancer, IBD, inflammatory bowel disease, pancreatic adenocarcinoma

## Abstract

Primary sclerosing cholangitis (PSC) is associated with hepatobiliary and colorectal cancers, but it remains uncertain if PSC increases the risk for pancreatic cancer. While some European studies have suggested an increased risk of pancreatic cancer in PSC patients, other studies have not. And these studies did not well account for presence or absence of concomitant inflammatory bowel disease (IBD). The purpose of this study is to investigate the prevalence of pancreatic cancer in United States veterans with PSC both with and without IBD.

**Methods:**

This retrospective study used International Classification of Diseases, Tenth Revision (ICD-10) codes to identify patients with PSC, IBD, and pancreatic cancer from the Veterans Affairs (VA) Corporate Data Warehouse. The prevalence of pancreatic cancer in patients with PSC only, IBD only, PSC with IBD, and neither PSC nor IBD were compared. Logistic regression was used to control for age, gender, chronic pancreatitis, diabetes mellitus, and tobacco and alcohol use.

**Results:**

A total of 946 patients with PSC were identified from a population of over 9 million veterans. 486 (51.4%) of these had concurrent IBD. Additionally 112,653 patients with IBD without PSC were identified. When adjusted for confounding factors, patients with PSC had a significantly higher prevalence of pancreatic cancer compared to the general population and those with IBD without PSC (2.4% vs. 0.2% and 0.5%, respectively).

**Conclusions:**

Veterans with PSC, particularly those without concomitant IBD, have a high prevalence of pancreatic cancer compared to the general veteran population. Our findings support the need for multicenter prospective studies investigating the benefits of screening for pancreatic cancer in patients with PSC.

## Introduction

Primary sclerosing cholangitis (PSC) is a rare disease characterized by intra- and extra-hepatic biliary ductal inflammation and obliterative fibrosis (1, 2). PSC is associated with significantly increased risk of colorectal cancer, cholangiocarcinoma (CCA), hepatocellular carcinoma, and gallbladder cancer (1–4). Although a few studies have shown an increased risk of pancreatic cancer in patients with PSC, the available data on the association between PSC and pancreatic cancer are mixed.

One of the first large studies identifying an increased risk of pancreatic cancer in patients with PSC was performed in Sweden. In this retrospective study of 604 patients with PSC, five had a diagnosis of pancreatic cancer, which translated to a 14-fold increased risk of pancreatic cancer versus the general population (5). However, this study did not control for the presence of IBD. Concomitant IBD is found in 54-100% of patients with PSC (6–8). Patients with IBD have more pancreatitis, a risk factor for pancreatic cancer (9, 10). Additionally, the importance of disease-specific effects is highlighted by the significantly increased risk of colorectal cancer among PSC patients with IBD in comparison to patients with IBD alone (3). A more recent Swedish study, which also did not control for concomitant IBD, identified 7 cases of pancreatic cancer among 1432 patients with PSC, showing a hazard ratio of 8 compared to the general population (95% CI 3.2-20.2) (11). Further, a Swedish study that did control for concomitant IBD found a cumulative probability of 2.3% of pancreatic cancer in patients with PSC-IBD, but only 0.5% in patients with IBD alone (12). However, several other European studies failed to reproduce these results; for instance, a study of 211 patients with PSC in the Netherlands and another study of 200 patients with PSC in Belgium did not find a higher frequency of pancreatic cancer in PSC patients (7, 13–16).

Data on the prevalence of pancreatic cancer in patients with PSC in the US population are scarce. A potential explanation is the rarity of PSC and the lack of a national registry of these patients in the US to allow for studies with large numbers of study subjects. The US Veterans Health Administration (VHA) is the largest integrated healthcare system in the United States and provides national data. The purpose of this study is to examine the prevalence of pancreatic cancer in United States veterans with PSC.

## Materials and methods

### Data source

Data were obtained from the VHA Corporate Data Warehouse (CDW), a centralized administrative and clinical data repository. The VHA is the largest integrated healthcare system in the United States and provides care to over 9 million veterans per year at 171 medical centers and 1113 outpatient sites of care across the country (17). VHA started transitioning to electronic health records in the 1980s and has since become almost exclusively electronic (18). Diagnosis codes compatible with the International Classification of Disease (ICD) are required to be associated any time a patient encounters the VHA for care, seeks VA reimbursement for care outside of the VHA, or has their medical history problem list updated. Health factors screening including alcohol and tobacco use occurs in a variety of settings including triage for outpatient encounters and inpatient admission. CDW search engines allow for identifying patients based on their ICD codes.

### Study population

Veterans 18 years of age or older whose records are contained in the CDW were included in this study. Patients with a diagnosis of PSC, IBD, and pancreatic cancer based on the International Classification of Diseases, 10th Revision (ICD-10) were identified. ICD-9 was not used for the study because PSC is not distinguishable from other forms of cholangitis by the ICD-9 code alone. IBD was restricted to ulcerative colitis and Crohn’s disease codes because unspecified IBD is not distinguishable from other unspecified noninfective gastroenteritis and colitis by code alone. Patients were divided into two major groups: those with PSC and those with IBD without PSC. The PSC group was found to have a bimodal distribution of pancreatic cancer based on the presence or absence of IBD, thus the PSC group was recategorized as two distinct populations based on the presence or absence of IBD.

### Study variables

Basic characteristics including gender, race, and date of birth were directly extracted as stored in CDW. Age was defined as patient age at the time of data collection or at time of death, as in the national VA database a veteran’s age stops increasing at time of death. The frequency of pancreatic cancer was assessed in all US veterans, patients with PSC, and patients with IBD. To adjust for other known risk factors of pancreatic cancer, alcohol and tobacco exposure were obtained from the health factors data and not based on the ICD-10 codes to prevent exclusion of patients that use less than an amount considered sufficient to associate with a diagnosis code. Any amount of alcohol or tobacco use, current or former, was included. Additionally, the presence of T2DM, chronic pancreatitis, other benign pancreatic neoplasms, extrahepatic CCA, pancreas transplant, and liver transplant were recorded (**Supplementary Table 1**).

### Statistical analysis

Multiple regression analysis was used to compare demographic and clinical features of the study groups, with all variables compared considered as factors except age. Two group differences for various factors were compared using Fisher’s exact test and odds ratios. Logit generalized linear models (GLM) were used to assess the statistical significance of differences between all four groups, using the R “stats” package, version 4.0.4 (19).

### Ethical considerations

The study was approved by the Institutional Review Board of the University of Maryland Baltimore and the Research and Development Committee of the VA Maryland Health Care System.

## Results

Of the approximately 23 million veterans with data in the CDW, 9,471,335 had at least one valid ICD-10 code (**Supplementary Table 1**). Across all screened records, 946 patients with PSC were identified, and 23 of these (2.4%) also had a diagnosis of pancreatic cancer. This prevalence is 10-fold higher than the general VA population with neither PSC nor IBD in which 22,513 pancreatic cancer diagnoses were found in 9,357,736 veterans (0.24%).

486 (51.4%) of the patients diagnosed with PSC also had a diagnosis of IBD. Additionally, 112,653 patients with IBD without PSC were identified, and of these 550 (0.49%) also had a diagnosis of pancreatic cancer. In patients with both IBD and PSC, 2 of 480 (0.42%) patients also had a diagnosis of pancreatic cancer, a rate similar to that seen in IBD patients without PSC (0.49%). Contrastingly, 21 of 460 (4.56%) patients with PSC without IBD had a diagnosis of pancreatic cancer.

**Figure 1:**
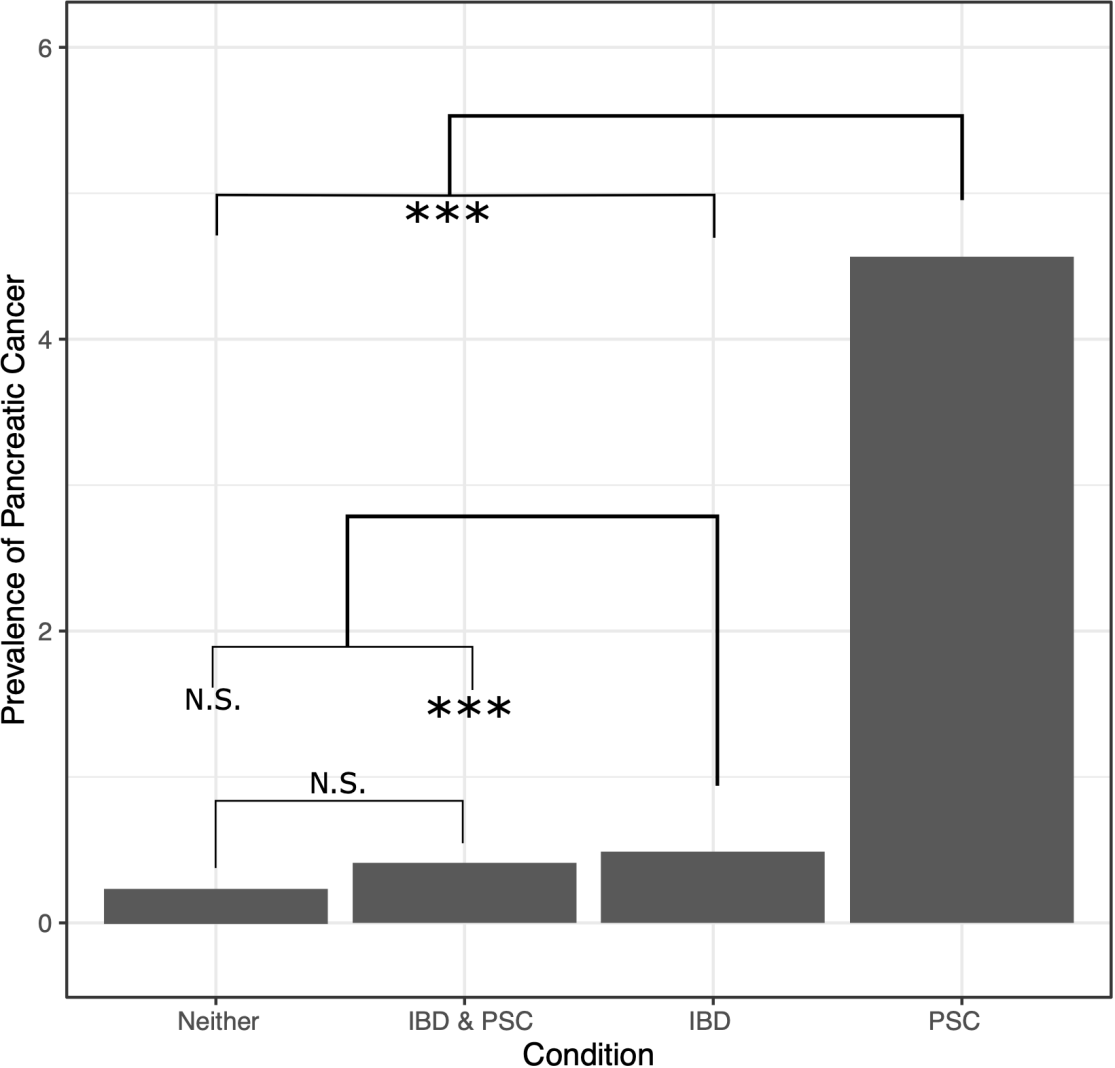
Prevalence (in population percentage) of pancreatic cancer in patients with neither PSC nor IBD, both PSC and IBD, IBD only, and PSC only. NS, not significant, *** = p-value ≤ 0.0075.

A univariate generalized linear model (GLM) was used to assess differences between groups and confirm that while patients with PSC and IBD and those with IBD without PSC had similar rates of pancreatic cancer diagnosis, patients with PSC without IBD had a much higher rate of occurrence (OR 9.75), while the general VA population had a lower rate (OR 0.49, p-value <2*10^-16^ for all regression coefficients; **Figure 1**; p-values of differences are in **Supplementary Table 2**). A similar GLM followed by least squared means was run on all factors to expand the analysis and distinguish which groups had significant differences across the factors assessed (**Table 1**; p-values of differences in **Supplementary Table 2**). There was no difference in race or rate of pancreas transplant across the four groups. And while some of the factors showed statistically significant differences, not all were significant in magnitudes, such as age and gender distribution. Chronic pancreatitis, other benign pancreatic conditions, liver transplant, and extrahepatic CCA were found to occur at much higher rates in patients with PSC with or without IBD (with no difference between them), and slightly higher rates in the IBD alone group than the general population. The general population and patients with both PSC and IBD had the lowest rates of tobacco exposure, followed by the PSC alone group, and the IBD alone group, while the IBD alone group was the only population to demonstrate an increased rate of alcohol use or dependency.

**Table 1:** Clinical characteristics of patients with: PSC only, IBD only, both PSC and IBD, and patients with neither disease.

	PSC Only (n = 460)	PSC and IBD (n = 486)	IBD Only (n = 112,653)	Neither PSC nor IBD (n = 9,357,736)
Patient Age	64.83 (IQR 55-76)	59.46 (IQR 48-72.5)	64.40 (IQR 54-75)	62.57 (IQR 49-75)
Male Gender	409 (88.91%)	454 (93.42%)	102,099 (90.63%)	8,101,665 (86.58%)
Race -White -Black or African American -Asian -American Indian or Alaska Native -Native Hawaiian or Pacific Islander -Mixed -No race listed	318 (69.13%) 101 (21.96%) 4 (0.87%) 2 (0.43%) 2 (0.43%) 6 (1.30%) 27 (5.87%)	355 (73.05%) 89 (18.31%) 4 (0.82%) 1 (0.21%) 1 (0.21%) 7 (1.44%) 29 (5.97%)	87,267 (77.47%) 16,206 (14.39%) 778 (0.69%) 658 (0.58%) 647 (0.57%) 1,494 (1.36%) 5,603 (4.97%)	6,213,661 (66.40%) 1,376,216 (14.71%) 101,425 (1.1%) 58,000 (0.62%) 64,014 (0.68%) 124,067 (1.33%) 1,420,353 (15.18%)
Tobacco exposure	343 (74.57%)	315 (64.81%)	91,457 (81.19%)	5,979,400 (63.90%)
Alcohol exposure	100 (21.74%)	104 (21.40%)	34,922 (31.00%)	2,138,779 (22.86%)
PCa	21 (4.6%)	2 (0.41%)	550 (0.49%)	22,513 (0.24%)
T2DM	177 (38.48%)	141 (29.01%)	36,289 (32.21%)	2,196,486 (23.47%)
Chronic pancreatitis	34 (7.40%)	22 (4.52%)	1,691 (1.50%)	38,720 (0.41%)
Other benign pancreatic conditions	34 (21.25%)	27 (5.56%)	1,238 (1.10%)	29,169 (0.31%)
Liver transplant recipient	55 (11.96%)	79 (16.26%)	303 (0.27%)	7,992 (0.085%)
Pancreas transplant recipient	0 (0%)	0 (0%)	7 (0.0062%)	309 (0.0033%)
Extrahepatic CCA	13 (2.83%)	12 (2.47%)	150 (0.13%)	4,176 (.044%)
Both PCa and CCA	2 (0.04%)	0 (0%)	36 (0.031%)	1,257 (0.013%)

**Figure 2:**
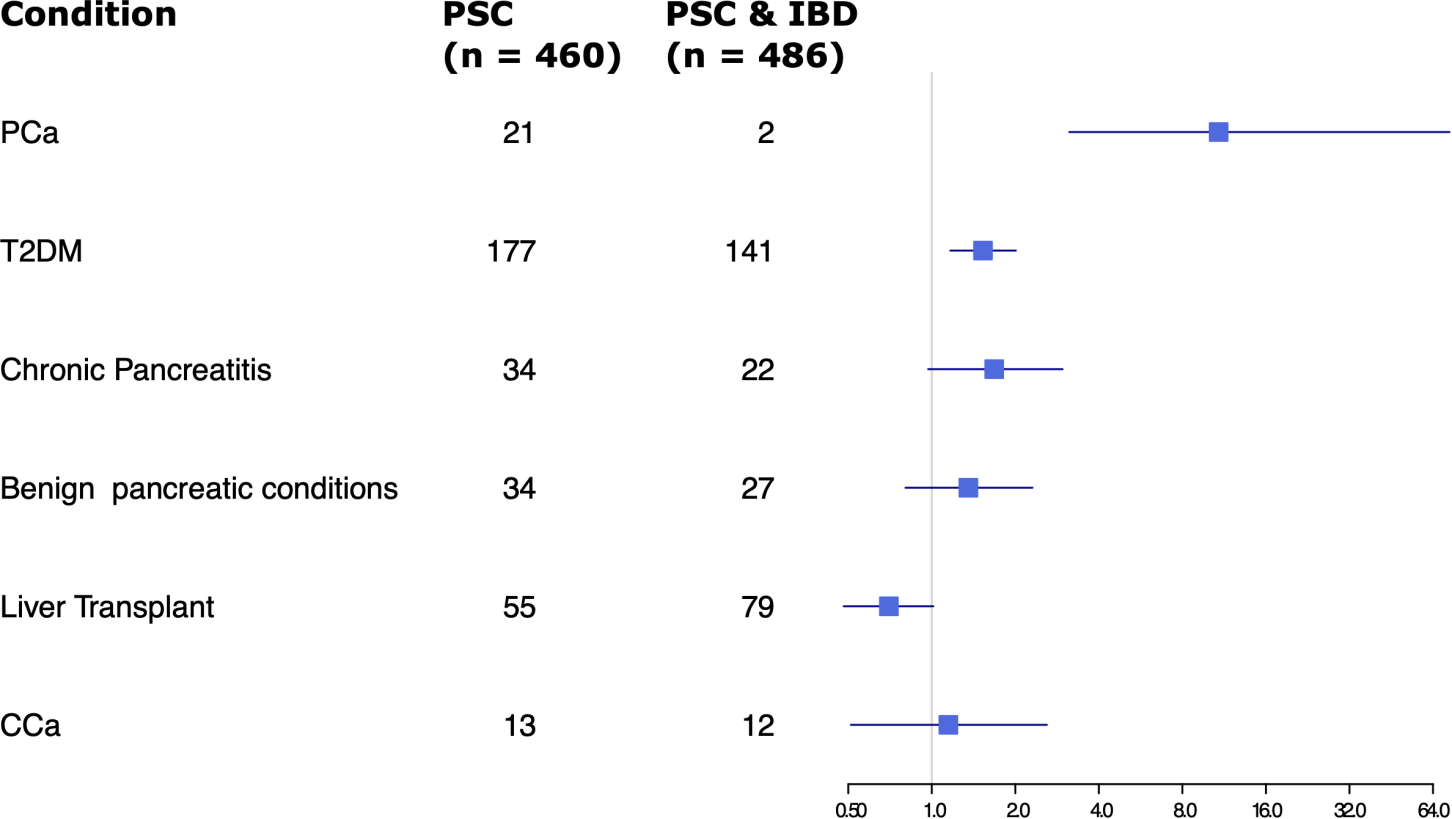
Forest plot of odds ratios for various factors assessed in patients with PSC only vs PSC and IBD.

Given the significant difference in rates of pancreatic cancer in the PSC alone group versus the PSC and IBD group, ORs of several factors were compared between the two groups, including rates of T2DM, chronic pancreatitis, benign pancreatic conditions, liver transplant, and CCA (**Figure 2**). Other than pancreatic cancer, only T2DM showed a statistically significant difference, with slightly increased odds in the PSC alone group.

Multiple logistic regression was used to assess how several factors came together to contribute to the risk of pancreatic cancer in these populations. Analyses were focused on those who had PSC, IBD, or both due to the computational limitations in performing logistic regression on millions of instances accounting for many factors. Among these three groups, those who had PSC alone, IBD alone, or PSC and IBD, age was found to have a small but statistically significant association with an increased rate of pancreatic cancer while chronic pancreatitis and diabetes along with male gender were found to have stronger associations. PSC patients were found to have an increased rate of pancreatic cancer independent of their history of chronic pancreatitis. Smoking history was also associated with an increased rate, while other benign conditions of the pancreas had an extremely strong association with increases in pancreatic cancer. Other conditions of the pancreas also had a strong association with PSC alone, suggesting the interaction between the two could be a contributing factor. Significant alcohol consumption and race did not appear to have an impact on the rate of pancreatic cancer (model details in **Supplementary Table 3**).

## Discussion

This large nationwide study shows that a diagnosis of PSC without IBD is associated with a markedly higher prevalence of pancreatic cancer compared to the general population (4.6% versus 0.24%, a nearly 20-fold increase). Although the rate of pancreatic cancer was found to be modestly higher among IBD patients compared to the general veteran population (0.49% and 0.24%, respectively), there was no significant difference in the prevalence of pancreatic cancer in patients with IBD without PSC compared to those with IBD with PSC (0.49% and 0.41%, respectively). As expected, known risk factors for pancreatic cancer such as T2DM, tobacco exposure, and alcohol exposure (9) were also examined and demonstrated significance in our study.

There are several possible explanations for higher rates of pancreatic cancer in patients with PSC. The biliary inflammation and obstruction that leads to cirrhosis and hepatobiliary malignancies may affect the distal common bile duct and result in reflux of bile acids, or possibly bacteria, into the pancreatic duct. The resultant pancreatic inflammation may be too intermittent or mild to cause clinically overt pancreatitis, but the chronic mild inflammation may be pro-neoplastic. In one study of 103 patients with PSC, 24% of the patients had pancreatic duct changes, which were associated with duration of disease and extrahepatic biliary involvement (20). Additionally, PSC may possibly affect pancreatic ductal cells directly - not as dramatically as to cause the same “beads on a string” appearance as is seen on a cholangiogram - but enough to cause chronic mild inflammation. Inflammatory cytokines have been shown to trigger inducible nitric oxide synthase causing DNA repair inhibition in CCA (21). The same enzyme was also noted to have increased expression in pancreatic adenocarcinoma (22). Pro-oncogenic mutations such as in tumor suppressor p16^INK4a^ and ErbB2 have also been noted in both PSC and pancreatic cancer (23–26).

Veterans are more likely to have exposure to substances such as Agent Orange, which increases the risk for various cancerous and non-cancerous conditions (27). However, the VA committee that monitors such associations has not found sufficient evidence to associate military service or chemicals of interest with pancreatic cancer (27). The prevalence of PSC in this study was 9.99 cases per 100,000 veterans. This is consistent with the prevalence of PSC among the general United States population. A study of Northern California residents found a prevalence of 4.15 cases per 100,000 and a study of Olmstead County in Minnesota found a prevalence of 13.6 cases per 100,000 (28, 29).

The results of this study showed that while patients with PSC and concomitant IBD and those with IBD without PSC had comparable rates of pancreatic cancer, patients with PSC without IBD had a significantly higher rate of pancreatic cancer. The findings of this study suggest that patients with PSC and concomitant IBD behave differently from those without IBD. Bowlus et al. examined liver transplant registrants and found that African American patients with PSC had higher Model of End-Stage Liver Disease (MELD) score, higher odds of requiring liver transplantation, lower age, less frequent concomitant IBD, and different human leukocyte antigen associations compared to European American and Hispanic American patients with PSC (30). In line with our study, their data support that PSC in patients without concomitant IBD may be clinically distinct from PSC in patients with IBD, and this difference may have a genetic basis. Why IBD appears to be ‘protective’ against pancreatic cancer in patients with PSC is unclear, but we theorize that anti-inflammatory effects of IBD-treatments, alterations in the microbiome of patients with IBD, or genetic differences between patients with PSC with IBD may contribute.

Our study found that IBD patients have a modest (two-fold) but significantly increased rate of pancreatic cancer compared to the general VA population. Patients with IBD have higher rates of acute and chronic pancreatitis, which predisposes them to pancreatic cancer (9, 10). Our study also found a higher rate of pancreatitis in the IBD group compared to the general VA population. Additionally, IBD patients tend to have more abdominal imaging resulting in potential detection bias, though this effect is expected to be small as pancreatic cancer rarely remains clinically silent.

Taken together, guidelines from the American College of Gastroenterology and the American Gastroenterological Association recommend routine screening for colorectal cancer, cholangiocarcinoma, and gallbladder cancer in patients with PSC, and screening for hepatocellular carcinoma in those with cirrhosis (31, 32). These guidelines include ultrasound as an option for hepatobiliary cancer screening. Conventional ultrasound may have reduced sensitivity for detecting pancreatic cancer due to the retroperitoneal location of the pancreas, presence of overlying bowel gas, and the body habitus of some patients (33). Currently, there are no recommendations for routine screening for pancreatic cancer in patients with PSC, which, if recommended, would likely be best accomplished through contrast-enhanced MRI or CT scanning. Routine screening for pancreatic cancer has proven ineffective in the general population but may be beneficial in higher-risk individuals (9). Although the results of this retrospective study are not sufficient to recommend routine screening for pancreatic cancer in patients with PSC, this study suggests an increased risk of pancreatic cancer in patients with PSC, particularly those without concomitant IBD, and supports the performance of future studies on the impact of pancreatic cancer screening in the PSC population.

## Limitations

Due to the relative rarity of PSC in the general population, the VHA CDW was used for data mining from over 23 million medical records. ICD-10 codes were used to identify the study population, which assumes the correct association of diagnosis and disease within the medical record. ICD-10 diagnoses of pancreatic cancer, PSC, and IBD were not confirmed by a more definitive chart review due to the magnitude of the sample size. Hence, this study is limited by the quality of the medical record and the diagnostic certainty at the time of documentation.

Distal CCA and pancreatic head cancer can have a similar clinical presentation and imaging appearance, so misdiagnosis is possible. Given the scope of our study, which relied solely on diagnosis codes, we were unable to verify the diagnosis codes through comparison with pathology. To address this concern, and to identify charts in which a provider may have added pancreatic cancer as a presumptive diagnosis before pathology results and then subsequently added the correct diagnosis of CCA, charts with ICD-10 codes for both pancreatic cancer and extrahepatic CCA were identified. Only two charts had both diagnoses in the PSC alone group and re-running the analyses assuming all charts with both pancreatic cancer and CCA were actually CCA-only did not change any of our findings.

We found that 51.4% of PSC patients had concomitant IBD, which is lower than the average of ~70% reported in other studies (6). There may have been some people who were prevented from entering the military due to preexisting IBD, which may have manifested earlier due to the different IBD phenotypes of PSC-IBD patients. Additionally, although the rate of concomitant IBD in our PSC cohort (51.4%) is lower than expected based on historical reports (8), a recent large cohort study in the UK, similar in scale to our study, found the rate of concomitant IBD in patients with PSC to be 54% (7). Readers should be aware that a misclassification in disease may have occurred in our retrospective study (and possibly in the UK study) that is based on diagnostic codes. It is possible that some veterans in our study with a diagnosis of PSC actually had secondary sclerosing cholangitis (SSC), such as IgG-4 related cholangitis, which is associated with autoimmune pancreatitis that can increase cancer rates. However, while autoimmune pancreatitis is associated with a number of cancers, it is not clearly associated with pancreatic cancer (34). Finally, the United States veteran population is largely male, resulting in 86.6% of our study population being male; as such, our findings may not be generalizable to a female population.

## Conclusions

Veterans with PSC, particularly those without concomitant IBD, have a higher prevalence of pancreatic cancer compared to the general veteran population and those with IBD without PSC. Although the results of this study are not sufficient to recommend routine screening for pancreatic cancer in patients with PSC, they highlight the need for large prospective cohorts to further investigate the risk of pancreatic cancer, and impact of screening, in patients with PSC.

## Data availability statement

The data analyzed in this study is subject to the following licenses/restrictions: They are veterans PHI. Requests to access these datasets should be directed to https://www.research.va.gov/programs/vinci/contact.cfm.

## Ethics statement

The studies involving human participants were reviewed and approved by University of Maryland, Baltimore IRB. Written informed consent for participation was not required for this study in accordance with the national legislation and the institutional requirements.

## Author contributions

BTS and EvR are responsible for the concept and design of this study. Material preparation and data collection were performed by AN, and statistical analyses were performed by MA. The first draft of the manuscript was written by AN and MA, and all authors contributed revisions and edits. All authors contributed to the article and approved the submitted version.
