# Supplementary material for: Primary sclerosing cholangitis and pancreatic cancer: A retrospective cohort study of United States veterans
Source: Front Gastroenterol (Lausanne). Author manuscript; Available in PMC 2024 Feb 12. (PMC10860374; doi:10.3389/fgstr.2022.1076788)
Supplement: supplementary tables [file NIHMS1963054-supplement-supplementary_tables.docx]

| Condition | ICD-10 Code | ICD-10 Name |
| --- | --- | --- |
| PCa | C25 | Malignant neoplasm of pancreas |
| PSC | K83.01 | Primary sclerosing cholangitis |
| IBD | K50  K51 | Crohn’s disease  Ulcerative colitis |
| T2DM | E9 | Type 2 diabetes mellitus |
| Chronic pancreatitis | K86.0  K86.1 | Alcohol-induced chronic pancreatitis  Other chronic pancreatitis |
| Other benign pancreatic conditions | K86.2  K86.3  D13.6 | Cyst of pancreas  Pseudocyst of pancreas  Benign neoplasm of pancreas |
| Liver transplant recipient | Z94.4  T86.4  Z48.23 | Liver transplant status  Complication of liver transplant  Encounter for aftercare following liver transplant |
| Pancreas transplant recipient | Z94.83 | Pancreas transplant status |
| Extrahepatic CCA^a^ | C24 | malignant neoplasm of biliary tract |

Supplementary Table-1. ICD-10 codes relevant to the study. ^a^ICD-10 code C22.1, malignancy of the intrahepatic ducts, was not included under CCA because it is unlikely to be misdiagnosed as PCa.

|  | PSC Only vs PSC-IBD | PSC Only vs IBD Only | PSC Only vs  Neither | PSC-IBD vs  IBD Only | PSC-IBD vs  Neither | IBD Only vs  Neither |
| --- | --- | --- | --- | --- | --- | --- |
| Age | **<.0001** | 0.9550 | **0.0385** | **<.0001** | **0.0009** | **<.0001** |
| Gender distribution | 0.0726 | 0.5885 | 0.4579 | 0.1549 | **0.0001** | **<.0001** |
| Race | 0.9283 | 0.9754 | 0.9584 | 0.7225 | 0.6857 | 0.4263 |
| Tobacco exposure | **0.0064** | **0.0018** | **<.0001** | **<.0001** | 0.9749 | **<.0001** |
| Alcohol exposure | 0.9993 | **0.0001** | 0.9410 | **<.0001** | 0.8705 | **<.0001** |
| PCa | **0.0054** | **<.0001** | **<.0001** | 0.9950 | 0.8724 | **<.0001** |
| T2DM | **0.0114** | **0.0220** | **<.0001** | 0.4341 | **0.0213** | **<.0001** |
| Chronic pancreatitis | 0.2507 | **<.0001** | **<.0001** | **<.0001** | **<.0001** | **<.0001** |
| Other benign pancreatic conditions | 0.6614 | **<.0001** | **<.0001** | **<.0001** | **<.0001** | **<.0001** |
| Liver transplant recipient | 0.2328 | **<.0001** | **<.0001** | **<.0001** | **<.0001** | **<.0001** |
| Pancreas transplant recipient | 1.0000 | 1.0000 | 1.0000 | 1.0000 | 1.0000 | 0.3483 |
| CCA [(extrahepatic)] | 0.9863 | **<.0001** | **<.0001** | **<.0001** | **<.0001** | **<.0001** |
| Both PCa and CCA | 0.9996 | **0.0019** | **<.0001** | 0.9998 | 0.9999 | **<.0001** |

Supplementary Table 2. P-values for differences in various clinical characteristics between the four groups.

|  | Beta coefficient | Standard error | Z value | P-value |
| --- | --- | --- | --- | --- |
| Intercept | -8.381404 | 0.663751 | -12.627 | < 2e-16 |
| PSC and IBD | -11.902167 | 177.717475 | -0.067 | 0.9466 |
| PSC | 1.99289 | 0.281709 | 7.074 | 1.50E-12 |
| Age | 0.029099 | 0.003671 | 7.927 | 2.25E-15 |
| Smoking exposure | -0.347065 | 0.119713 | -2.899 | 0.00374 |
| T2DM | 0.515766 | 0.09026 | 5.714 | 1.10E-08 |
| Chronic pancreatitis | 0.844349 | 0.096136 | 8.783 | < 2e-16 |
| Other benign pancreatic conditions | 1.183341 | 0.142451 | 8.307 | < 2e-16 |
| Alcohol exposure | 3.003553 | 0.113917 | 26.366 | < 2e-16 |
| Asian race | 0.06947 | 0.092888 | 0.748 | 0.45453 |
| Black or African American race | 0.510675 | 0.750407 | 0.681 | 0.49617 |
| Mixed race | -0.115623 | 0.600238 | -0.193 | 0.84725 |
| Native Hawaiian or other Pacific Islander race | -0.30165 | 0.723928 | -0.417 | 0.67691 |
| No race reported | -0.746026 | 0.938386 | -0.795 | 0.42661 |
| White race | -0.150686 | 0.626452 | -0.241 | 0.80991 |
| Male Gender | 0.519733 | 0.216256 | 2.403 | 0.01625 |
| Interaction between other pancreatic conditions and IBD/PSC status (relative to IBD alone) | 11.263352 | 177.719104 | 0.063 | 0.94947 |
| Interaction between other pancreatic conditions and PSC only status (relative to IBD alone) | -1.459568 | 0.536449 | -2.721 | 0.00651 |

Supplementary Table 3. Model details of the multiple logistic regression.
